# Ohio College of Dental Surgery

**Published:** 1889-04

**Authors:** 


### PROCEEDINGS.

### Proceedings of Dental Surgery. Stockholders Meeting of the Ohio College, March 5th, 1889.

The second annual meeting of the Stockholders of the Ohio College of Dental Surgery was held in the lecture room of the college building at 3 P. M. on the above date. On motion of Dr. J. I. Taylor Dr. W. H. Sillito was elected Chairman, and Dr. C. I. Keely, Secretary of the meeting.

The minutes of last meeting was read and approved. On motion of Dr. J. Taft a committee of four was appointed to look up the matter of stock, to find out as far as possible how many shares are in force and the location of them. The chair appointed on this committee Drs. J. I. Taylor, J. Taft, H. A. Smith, and Jas. Leslie. On motion of Dr. H. A· Smith a committee of two, consisting of Drs. J. I. Taylor and F. H. Hunter, were appointed to confer with the college faculty in regard to sinking fund for the college.

On motion of Dr. F. H. Hunter the hour of meeting was changed from 10 A. M. to 3 P. M. Dr. H. A. Smith, dean of faculty made the following report:

*To the Stockholders of the Ohio College of Dental Surgery.*—The Faculty are pleased to report the forty-third session has been, in some respects, the most successful in the history of the college. The number of matriculates has been greater than any previous year. Total number 147; seniors 67; juniors 80.

When we consider the standard for admission is being made higher each year the steady increase in number of students shown for past ten years we think, clearly indicate the college is gaining in reputation. The examination by the professors in their respective departments shows that the didactic teaching is abreast with and fully as efficient as in any previous year.

It is with especial pride we call your attention to the extent and character of the clinical teaching during the past session. The infirmaries have been crowded beyond their utmost capacity and in the variety and character of the operations we may safely invite comparison with any similar institution.

Our demonstrators have beeen selected with especial reference to their fitness for their duties. Acting on the principle that "The laborer is worthy of his hire" we have paid liberal salaries and thus have been able to command the best energies of our efficient corps of teachers.

We have this year more than formerly drilled the junior students in operating and mechanical teachings, that is, requiring the students to perform as far as practicable all operations out of the mouth; the training of the hand and eye thus acquired has power to be of great assistance to the student when required to make operations in the mouth.

In conclusion we wish to call your attention to the great need of larger accommodation for students both in the teaching and clinical departments. We have been greatly hampered by lack of room the past year. Just how this may be overcome we are not prepared to state; we rather leave it to your better judgment.

On motion the report was received and ordered spread on the minutes. On call for stock it was ascertained that fifty-one shares, a majority, was represented and on motion the election of three trustees to serve for three years, and one to fill the unexpired term of Dr. G. W. Keely, deceased, was entered into which resulted in the election of Drs. J. I. Taylor, F. H. Hunter, and H. A. Smith, for the term of three years, and Dr. C. I. Keely, for the unexpired term. On motion adjourned till 3 P. M. on the Tuesday preceding the first Wednesday in March, 1890. CHAS I. KEELY, Secretary.

---

A TRUE physician has no professional secrets. If he discovers a new remedy he makes it known to the whole profession; he who tries to conceal a means of relief loses cast and is liable to expulsion. In treating a patient his own pecuniary interests are always a secondary matter. The welfare of the patient outranks all other considerations in the mind of a good physician. That is the standard; and the fact that some members do no act up to it only proves the weaknes of human nature. The standard itself is higher than that of the trader.—*Med. Herald.*

